# Conflicts and conflict regulation in hospices: nurses’ perspectives: Results of a qualitative study in three German hospices

**DOI:** 10.1007/s11019-012-9459-8

**Published:** 2012-12-23

**Authors:** Andreas Walker, Christof Breitsameter

**Affiliations:** Lehrstuhl für Moraltheologie, Katholisch-Theologische Fakultät, Ruhr-Universität Bochum, Universitätsstraße 150, 44801 Bochum, Germany

**Keywords:** Conflict regulation, End-of-life decision making, Ethics of care, Hospice, Palliative care, Terminal care

## Abstract

The present article considers conflicts and conflict regulation in hospices. The authors carried out a qualitative study in three hospices in North Rhine-Westphalia, Germany, to explore how conflicts arise and how conflict regulation proceeds. Hospice nurses should act according to a set of ethical codes, to mission statements of the institution and to professional standards of care. In practice the subjective interpretations of codes and/or models concerning questions of care are causes of conflicts among nurses, with doctors, patients and family members. The management has two choices to react to these conflicts. It can either tolerate the conflicts, as long as they do not disturb the daily routine. Or it can increase the degree of organisation by integrating the different viewpoints into its own program and/or by restructuring its organisational units.

## Introduction

Hospices in Germany are divided into various hierarchical levels. In addition to the director of the hospice, there is a director of patient care and the nurses who are employed either full or part-time. Furthermore, some hospices employ a spiritual care giver and/or a chaplain, a social worker and a coordinator for the voluntary staff. Hospices in Germany are small (8–10 beds), which is why they rarely employ doctors trained in palliative care or general practitioners as permanent staff. It follows that nurses have the most intense and most time-consuming contact with the patients when it comes to meeting their medical, social, psychological and spiritual needs.

Ideally, hospice nurses will act according to a set of ethical codes which have been developed especially for this profession. According to the ICN “Codes of Ethics for Nurses”, hospice nurses should endeavour to create an environment “in which the human rights, values, customs, and spiritual beliefs of the individual, family, and community are respected” (ICN 2006, p. 2). The guidelines embedded in these ethical codes allow the nurses some room for interpretation, which constantly needs to be renegotiated.^1^ With regard to medical issues, nurses regularly consult professional physicians; they discuss issues concerning nursing with their colleagues, social and spiritual needs with a spiritual care giver, a chaplain and/or volunteers. The ability of patients in the latter stages of terminal illness to take their own decisions is often severely limited, leaving the hospice nurses to take decisions regarding a patient’s ongoing treatment together with family members. It is equally challenging for nurses to manage a patient’s privacy and intimacy, because as the process of physical debilitation advances, so the vulnerability of the hospice resident increases.

How and when the rules embedded in the ethical codes are to be interpreted, however, is not only determined by the physical and mental state of the patients, but is also influenced by the position of the nurse within the institution. Nurses in Germany often have a special education or a special area of responsibility; some nurses are trained as geriatric nurses. If they have additional training, they are responsible for certain forms of therapy, such as aroma or music therapy. Further training may lead to a qualification as a case manager or a director of patient care, a specialist in palliative care and/or spiritual care. As a rule, hospices have a system of primary nursing, whereby a nurse is given primary responsibility for a resident. As a result of these various functions and areas of responsibility, there are differing ways in which nurses can tailor their care to the needs of their patients.

The bulk of the literature discusses the various ethics of care from a theoretical point of view (Fry 1989; Volker 2003; Edwards 2009, 2011, Nortvedt et al. 2011) with few practical references. Decision-making models are also examined within a theoretical framework (Greipp 1992; Tarlier 2004). However, little work has been undertaken to reveal how the “norms” laid out in the ethical codes and the ethics of care currently function in practice (cf. Heikkinen et al. 2006; Jonasson et al. 2011). We therefore considered it worthwhile to examine whether and to what degree the room for interpretation in decision-making leads to differences of opinion and conflicts, and what consequences the conflicts have. In this case, the term “conflict” should not be taken to mean an individual mental process in the sense of “simultaneous opposing tendencies within the individual to accept and reject a given course of action” (Janis and Mann 1977, p. 46). For the purposes of our research, “conflict” is taken to mean contrasting, mutually exclusive positions between two parties that become openly apparent.^2^

This approach has given rise to the following two research questions:

- What conflicts arise in hospices during the daily work routine between nurses, with doctors or general practitioners, with the patients and their family members?
- What strategies are there to settle these conflicts or to regulate them?

## Methods

The research questions just mentioned required a qualitative design, with data collection being predominantly based on observations and interviews. The study was conducted in three hospices catering to inpatients and run by independent service providers in North Rhine-Westphalia. Data collection and evaluation was based to no small degree on the methods of *grounded theory* which was developed by Glaser and Strauss (1967) and modified by Juliet Corbin and Strauss (2008). After the relevant Ethics Commission had raised no objections following the submission of the study protocol,^3^ between October 2010 and November 2011 one of the authors accompanied and observed the work of the nursing staff during *patient handovers*, *team meetings* and *case conferences*. The intention behind observing the meetings was to become better acquainted with the field of research and to gain a general understanding of the kinds of challenges with which the nurses were being confronted in their everyday work. Those present at the patient handovers were the nurses on duty; the case conferences were attended by all the nursing staff responsible for the particular patient; the team meetings involved, whenever possible, the entire nursing staff. Two of the hospices worked with palliative care doctors who regularly took part in the patient handovers.

Additionally, between November 2010 and November 2011, a total of 17 semi-structured interviews^4^ were conducted with full-time hospice staff, including nurses, the directors of patient care and the directors of the hospices. Nurses from the various hospices belonging to different age groups were included in the study to meet the requirements of ‘theoretical sampling’. Because nurses are primarily women, care was taken to try and include male nurses in the questioning in order to gain a varied image of our object of research. Fourteen interviewees had trained as nurses and three had trained as geriatric nurses. All except two of the interviews took place in the respective hospices. The interviews were set up using thematic guidelines which were based on the observations. The initial question was directed at the interviewee’s training and his or her motivation for working in a hospice. Subsequently, the interviewer raised the topics of conflict situations and the question of conflict resolution and regulation. Additionally, the staff members were asked about the degree of latitude available to them when taking decisions, about their interaction with the residents and their family members, and finally about communication and documentation. If appropriate we referred to issues raised at the different staff meetings.

In the course of the study the various levels within the hierarchy of the hospices were taken into consideration in order to gain an insight into those research questions which had remained unanswered. The findings from the interviews were compared to the recordings made during the observations and supplemented with further observations. This process sometimes gave rise to new questions which were then addressed in the subsequent interviews. We conducted several such cycles of data collection, evaluating the material as we went along. The categories which were established during the research were processed according to their theoretical characteristics and once again tested in the field. The encoding process was conducted by two participating researchers with differing background specialisations working independently of one another. The results were compared and discussed in regular meetings. The process of data collection was ended after it had been established that no further categories were to be identified in the observations and interviews, and it could be said, in relation to the underlying research questions, that a state of theoretical saturation had been reached.

In what follows, the main findings of the study will be presented. The quotes are related to textual material taken from the interviews; “M 1”, “M 2” etc. indicates statements by the full-time staff. Indexation was carried out according to the chronology of the recordings.

## Results

Conflicts were classified differently by the staff members according to their level of gravity. What was understood by one person to constitute a conflict was understood by another to be a difference of opinion, part of the normal process of discussion or part of an atmosphere of constructive debate. Independently of the differentiated perceptions of conflict, various patterns of conflict became apparent which we have divided into *areas of conflict* and *conflict constellations*. (1) *Areas of conflict* are related to substantive questions and incorporate matters related to *medication* and *care*. (2) *Conflict constellations* are related to social interactions and incorporate (a) *the relationship between the nurses and the patients*, (b) the *relationship between the patients and their family members as viewed by nurses* and (c) the *relationship between the nurses and the family members*. *Areas of conflict* and *conflict constellations* are categories which inevitably have some overlap,^5^ since an *area of conflict* presupposes forms of social interaction of nurses with patients, family members and doctors. This becomes particularly apparent in the context of “care” and in the context of “medication”. Nevertheless, the categories certainly possess a distinct characteristic of their own. An area of conflict usually centres around technical knowledge, whereas a conflict situation is the result of dealing with social situations. In addition to the conflicts themselves, we were also interested in how and whether the conflicts are resolved or regulated. We divided (3) *conflict regulation* into (a) *conflict regulation within areas of conflict* and (b) *conflict regulation of conflict constellations*.

### Areas of conflict

#### Medication

One of the hospices we observed works together only with different general practitioners, the others work together with one or two doctors who are specialized in palliative medicine and sometimes with the general practitioner of the patient. Neither of these doctors or general practitioners is present permanently. They come once or twice a week to the hospice or in case of emergency. They have the responsibility for the medication in general; however the dosage of the medication from day to day lies in the hand of the nurses.

Due to the variety of experience that the care staff has with medications, there is a danger of conflicts arising when medications are being administered. Nurses tend to have a certain amount of latitude in assessing the dosage of a particular medication once the doctor has issued the relevant instructions and can, in the event of a certain medication being ineffective in dealing with a specific symptom, select an alternative—providing that the doctor has compiled a list of appropriate medications. As a rule, the selection of the medication is agreed with the patient, as long as they are capable of doing so. Nevertheless, opinions often diverge amongst members of the team as to whether a patient is experiencing pain or not and whether a particular type of medication should be administered, despite its undesired side-effects, or not. This becomes especially clear when nurses are dealing with psychotropic medications.

So in our day-to-day work at this hospice, psychotropic medications are frequently a problem. For example, when Tavor^6^ is being administered, the willingness of the nurses to use it is really different. One might say: ‘My God, he’s suffering so I’ll give him some.’ Another might say: ‘No, why should I? I’m going to let him remain conscious for a while and cope with it.’ (M 6, 323–328)

In respect to the administering of stronger medications causing drowsiness, one of the interviewees stressed the positive aspect of having to deal with his colleagues:

For example, when I get the feeling that I should actually be giving the patient some form of medication at that moment, which might help him on the one hand but on the other hand might make him sleep more and put him in a state of semi-consciousness. At that moment, I don’t know myself: is that advisable? Is it the right thing to do? Then I talk it through with my colleagues, if I can’t talk to the patient about it. These are really important things that happen to us. They keep leading to discussions, sometimes to arguments. But I think it’s important to have a culture of constructive debate at our workplace. (M 1, 341–349)

Differences of opinion regarding medications also arise between the team and the doctor (cf. Frederich et al. 2002). The source is, for instance, the dosage of the medication or—from the perspective of the team—the premature discontinuation of medication. Especially if the hospice works together only with general practitioners this can lead to additional difficulties.

What I don’t like so much is being given instructions by someone who isn’t present, like down the phone. […] It happens and makes me a bit uncomfortable. If a family doctor has taken over here and doesn’t do hospice visits all that often or come by on his rounds very much. […] I don’t think that’s satisfactory. (M 15, 407–413)

This particular nurse complained that many general practitioners have hardly any experience with palliative medicine and that they would prescribe medication that have no effect whatsoever due to the disposition of the patients, or that doctors sometimes fail to provide accurate information about dosages. But differences are also sometimes caused by the doctor adopting a paternalistic stance towards the patient, something to which the care staff reacts with great sensitivity since they believe the autonomy of the patient is paramount.

#### Care

Fundamental differences in the way in which the patients receive care tend to arise because the nurses have differing opinions regarding what they think the resident wants or needs. Some nurses fail to show an appropriate level of calm. This has an effect on the time-management of the hospice, as one of the directors of patient care reported:

What does it mean to work in a hospice? Lots of people think it’s like this: preferably being there for the patient twenty-four hours a day. And very often it’s not even what the patient wants, […] They are actually pretty happy to have a bit of peace and quiet. […] The care staff would probably want to take over all the jobs, even the everyday support of the patients which is really the most fundamental task of the volunteer staff. But the nurses think they can keep control of everything but then they find they can’t cope. […] I think that causes a lot of conflict here. (M 6, 109–125)

The interviewees said that the ability to maintain a sense of calm and composure and a willingness to accept a “no” from the patient constituted the right attitudes to care in a hospice. Hence, many “points of friction” (M 1, 312) arise from the individual characters of the nurses, who interpreted the patients’ wishes in different ways.

There are staff members here who think that every resident has to be washed from head to toe every day. And then there are others who don’t think that’s so important. They tend to focus on something else and say: “On one day I’ll do a bit less, and instead do a nice foot massage or a back massage or I’ll just sit down and hold their hand […].” But within the team these things are seen differently. (M 12, 89–97)

Such differences do not necessarily lead to conflicts, but they can if a patient’s “no” is ignored. One director of hospice explained: “As a hospice it is our task, or as a hospice movement, to ensure that the wishes and needs of the dying are always at the forefront of what we do” (M 14, 29 ff.). More than in other facilities it is vital that the individual nurse assume responsibility for patient care and respects that the “terminally ill person knows all too well what is good and right for him” (M 14, 36 f.). There is often some confusion on the part of the nurse over what constitutes the needs of the patient and what the nurse feels she should do to benefit the patient. In two of the three hospices, some nurses’ paternalistic approach was presented to us as a phenomenon that repeatedly lead to arguments and conflicts within the team.

In the hospice there are definitely lots of differences of opinion about what somebody thinks the patient might still want. And I think that my colleagues tend to interpret quite a lot of their own ideas into that. Or family conflicts that absolutely still have to be settled, or so some colleagues think. They just can’t leave it unaddressed. It’s all about leaving things to the patients, and it’s about dignity and it’s also about taking the patients seriously. And that’s not always the case, it seems to me. (M 5, 43–50)

Some of the care staff would allow the patient to sleep on despite it being time to administer their medications, whilst others would wake the patient up to administer it, according to one interviewee. Such divergent positions could lead to lively and productive discussions within the team. On the other hand, the differing interpretive approaches also bore potential for conflicts.

### Conflict constellations

#### Patient–nurse

Generally, the interviewees answered the questions as to whether there were any difficult patients in the negative. In answer to the question as to whether there were typical hospice patients, the answer was also negative, since each one was different. If patients do not cooperate with their care, try to insist on having their own way and are “clearly able to say ‘no’” (M 6, 596 f.), this is often seen as a challenge which is not always very pleasant for the nurses. Some patients use bad language and are unhappy, which can present an excessive challenge to a nurse. Patients like this “eventually end up in a case conference” (M 6, 602). With patients who prove to be resistant to the recommendations of the nurses, it can happen that they pose a danger to themselves or others. The team then has to consider the degree to which it is appropriate to intervene in the autonomy of the patient.

Differences can also arise between the patient and the nurses when medications are being administered. It may be problematic for the nurses if a patient prefers to endure his pain and does not wish to take any medications to alleviate it. On the other hand it is also difficult when patients no longer wish to remain conscious and request strong sedatives.

#### Patient–family member as viewed by nurses

In the context of conflicts arising between patients and their family members, we were particularly interested in what form any intervention by the care staff might take. The nurses have differing perceptions about whether to intervene in a conflict between a patient and a family member.

[This] whole complex of terminal care […] where we have staff there who say: “This should definitely be dealt with.” And then there are others there who say: “We have no right to become involved. We just have to let it be.” So it’s somewhere between these two positions then. (M 12, 40–47)

The three hospices in our study made the following provision for family members: in one hospice most of the patients’ rooms are positioned adjacent to a further room where a family member can spend the night. In this case, a clear decision has been made to define the “challenge of hospice care” to include “working with family members” (M 13, 1, 58 f.). One of the other hospices had only one room for family members. The essential idea of hospice care envisages family members being integrated into the process of terminal care. The more frequently they spend time in the hospice themselves and stay the night there, the closer they and their difficulties in accepting the death of the patients are to the care staff, who then find themselves having to provide care for them too. This is an additional burden for the staff, especially when family members make sizeable and often time-consuming demands on the personnel.

Situations sometimes arise where staff members are given the impression that the patients might need protection from their family members who might be threatening to overwhelm them by demanding more from them than the patient is able or desirous to give. Nevertheless, as long as the daily care routine and the hospice are not affected by a conflict between a patient and a family member, the nurses tend not to intervene. This is true in most cases, except when a family member is unable to cope with the patient’s terminal deterioration and specific spiritual care skills are required to integrate the family member into the process of terminal care and to provide care for the family member too.

#### Care staff–family member

Conflicts often arise between the care staff and family members when the patients have not been informed where they are or where they are being taken, and the family members continue to request that the patients are not informed of their location in the future. This was a situation mentioned repeatedly in the interviews. This is all the more problematic when the patients are required to provide advance consent to entering a hospice.

The legal requirements in Germany alone stipulate that a hospice must explain to its patients what is happening to them, if this has not been done beforehand. But it is also the case that the hospice’s understanding of its duty of care precludes the possibility of the family member’s requests or wishes being granted.^7^

For us, it’s a prerequisite that the people who come here know where they are. That means they are not in a sanatorium and not a small hospital, but that this is a hospice. There are some family members who simply say: “Don’t tell them.” That is just not possible. […] You know, we don’t go around pointing it out all the time, but we do try and bring up death and dying as a topic of conversation from time to time. (M 7, 260–266)

At the same time, however, situations in which the family members refuse to accept, to some extent, that the patient is about to die, are seen by the care staff as being difficult to handle. This is especially true in cases where the patient is suffering from brain metastases or dementia, cases which therefore mean that it is not possible to establish beyond doubt whether the patient knows his whereabouts. However, it was most often the case that the patient was very much aware of what a hospice meant for him, as the interviewees repeatedly stressed—even if the family member was trying to protect the patient as well as himself by telling a lie. It is possible for a family member to sustain this kind of self-defence mechanism for the entire duration of the patient’s stay in the hospice.

The family member will only hear what he wants to, and only see what he wants to see. And that is of course only human but it puts you as a member of the care staff in a really difficult position. (M 9, 268–271)

Questions regarding nutrition or provision of liquids also present a challenge to the care staff when communicating with family members, because the latter often insist on the continuation of the patient’s previous diet—even when continuing it would mean exacerbating the suffering of the patient rather than alleviating it.

### Conflict regulation

During our observations, we were unable to identify a situation in which conflicts were resolved in the sense that all conflicting positions had disappeared. With this in mind, it seems to us to be more appropriate to adopt a piece of terminology from Ralf Dahrendorf, and to speak of conflict regulation (Dahrendorf 1972, p. 41).^8^

#### Conflict regulation within areas of conflict

Differing opinions concerning *medications* repeatedly lead to discussions among the care staff or between nurses and the doctor responsible for the patient. In this context, it can happen that the doctor, because he carries ultimate responsibility, will use his position of authority to determine a course of action, but he can also coordinate with the team and rely on their experience. Even if the wishes of the patient and his general welfare are at the forefront of decision-taking regarding the issue of medications, a conflict between the doctor and the team cannot always be resolved satisfactorily for both sides. In order to regulate conflicts like these more efficiently, two of the hospices in our study follow a strategy of involving the doctor in the patient handovers. The aim is that the he gets to know to some degree the daily work routine of the hospice. The third hospice invites general practitioners to conceive their work in the hospice as an opportunity to acquire additional training in palliative care and, in doing so, to better understand the perspective of the care staff.

Conflicts which arise between members of the nursing staff concerning care often continue even after they have been addressed, because nurses fall back into their old individual behavioural patterns. We did not observe any cases in which infighting emerged from these differences of opinion. However, differences of opinion did partially determine the discussions during the daily work routine and returned as topics of conversation during the patient handovers, case conferences and team meetings. According to one of the directors of hospice, any discussion that takes place must never lose sight of “what are the needs of the dying person, and not what are our needs or what are my own needs” (M 14, 42 f.). In this regard, it was possible for us to observe how the three hospices in our study pursued varying policies concerning these differences of opinion. In one hospice, such issues were discussed openly in the team meetings; in another, the issues were mentioned to us rather than being openly discussed. The strategy for finding a solution, however, consisted of the team agreeing on a common framework for action by which everybody agreed to abide, or the director of hospice mediated between the staff members or supplied a framework for action themselves. A further strategy for dealing with differing opinions regarding care was to remind the nurses of the mission statement or to suggest a revision of the mission statement in consultation with the team. By doing this, it was hoped that greater consensus might be reached within the team regarding the implementation of care, if everybody had contributed to formulating a new mission statement.

#### Conflict regulation within conflict constellations

Members of the care staff may relapse into their customary, paternalistic patterns of behaviour, often urging the patient to eat or drink something although he has expressed a clear “no”. If the patient feels as though the nurse’s behaviour is becoming overly insistent, it will be discussed within the team, although this doesn’t necessarily mean that the problem is actually solved. The problem might continue to exist even after several rounds of discussion.

If a nurse who is charged with primary responsibility of care cannot cope with a certain patient, then that nurse can pass on that area of responsibility to someone else. If such a problem exists not just with one nurse but with several nurses or with the entire team, then that specific patient will become the subject of a case conference. This does not necessarily mean that the conflict is resolved; nevertheless, the team usually agrees on a strategy for coping with the situation.

If a patient refuses pain therapy because he is concerned of losing decision-making capacities, than a discussion could be held between the nurse and the patient and possibly with family members too, in order to find a solution to the pain issue which is satisfactory for the patient. In general, the nurse knows from experience that a specific dosage of the medication could improve the patient’s quality of life. This does not mean, however, that an agreement is reached between the patient and the nurse. Occasionally, the patient will still refuse the analgesic medication even after a discussion has occurred to explain the side effects.

In a conflict between a patient and a family member, nurses are usually at liberty to decide what action to take, as long as the conflict is not negatively influencing the care or causing undue stress to the patient. In one case, a woman felt that her husband was putting her under pressure and entrusted a nurse with her feelings. The nurse suggested that she no longer saw her husband in her own room but in a common room, thus solving the problem. But it is frequently the case that conflicts are much harder to resolve. If the nurse is unable to solve the problem independently, then the hospice’s director of patient care will be consulted. In the case of a medical problem, nurses can turn to a doctor for advice. However, when confronted with serious cases it was also of great help, as our interviewees stated, to be able to rely on spiritual or psychological care and not to leave the nurses with the main responsibility for mediating between the patient and the family member. If nurses feel they are not making any progress in their work with family members, then it can sometimes be of help for them to engage in a discussion with the director of hospice, in order to reflect on their own conduct. One of the hospices in our study held an internal workshop on this topic in which many of the nurses’ difficulties were raised, and solutions to them were sought. This was a great help to the care staff regarding their everyday duties.

Nurses will intervene in an argument between a patient and a family member if it escalates and is a disturbance to other patients. In such cases, if nurses are unable to calm the situation, then the director of hospice will become involved. Intervention is also necessary when it becomes impossible to conduct the basic work routine of everyday care, and the nurse is being put under pressure by a family member. In the case of intra-familial problems, consideration must be given to whether intervention actually serves in a constructive way towards finding a solution, or whether the conflict is a purely private matter.

In conflicts between the staff and family members which are centred on informing the patients about what is happening to them, the family members are approached and encouraged to participate in the process. The care staff will specifically address the topic of death in order to establish the degree to which the family members themselves have come to terms with the impending death of the patient. This does not mean, however, that the family member is always successfully integrated into the terminal care. It is not uncommon for the family member to continue to deny the imminent death of their relative. Similarly, regarding the question of the patient’s provision with solids and liquids, a nurse will seek to convince the family member that continuing to provide food would be counterproductive.

## Discussion

According to our empirical research regarding *conflict constellations* and *areas of conflict*, two explanatory models can be used to schematically classify the ways in which conflicts are handled, namely: (a) *Conflicts from the Role Perspective* and (b) *Conflicts from the Structural Perspective*.

### Conflicts from the role perspective

George Herbert Mead identified the “social act” as being integral to cooperation between individuals. In order for individuals to be able to work together, they must be willing to accept each other’s attitudes and desire a common outcome. This outcome is achieved by the individual’s willingness and the potential for him to take on “the rôle of the generalized other” (Mead 1959, p. 87). Conflicts arise when, in trying to achieve a specific outcome, the role-taking fails (Mead 1967). Finding the solution to a problem was therefore dependent on improving the individual’s role-taking abilities.

In order to improve an individual’s abilities in “taking the role of the other”, a hospice will try, within the discussion processes, to find solutions to existing conflicts that are related to the aims, values and symbols (cf. Rössel 1999, p. 35) of a hospice as an institution. These values, aims and symbols give particular consideration to the well-being of the patients, and the ideal of a dignified death. According to Mead, we might expect the nurses to correct their behaviour through dialogue, if it has been criticised.^9^ However we were repeatedly informed that the conflicts that arose from the *individual interpretations* of what constituted appropriate care were not resolved, but continued to exist even after a discussion had taken place.

Lonnie Athens has modified Mead’s model to allow for such problems, thereby achieving a higher degree of descriptive adequacy. He differentiates between institutional social acts which are defined by prescribed sets of rules to which all parties are subject, and emergent social acts within which behavioural maxims are developed during the act itself. In addition, Athens has added the aspect of power to the social model: “dominance is an inescapable element of all complex social acts” (Athens 2002, p. 31; cf. McCarthy 2010). The flatter the hierarchy, the easier the process of “taking the role of the other” is for the individual. But even in situations where hierarchies are kept fairly flat, such as in a hospice, the relative positions of the staff members to each other retain an element of asymmetry due to the roles assigned to each nurse (e.g. primary responsibility of care) and each nurse’s individual level of knowledge and experience (for example, some nurses may have particular expertise in the fields of music or aroma therapy). These *different competences*, i.e. “information control” (Collins 1975, p. 310), could explain why the act of “taking the role of the other” is unsuccessful.

In summation, the social act, as it exists in a hospice, is subject to rules (mission statements, care directives). Nurses will feel that a bond has been created between the hospice and themselves through this normative framework of control and the nurse’s identification with the aim of ensuring the wellbeing of the patient (cf. Tadd et al. 2006; Verpeet et al. 2006; Numminen et al. 2009). Our observations revealed that although the nurses tried to base their work on the mission statements, they had to be consistently reminded of them. One of the behavioural ideals laid down in the rules, a “transfer of attitudes”, often failed to take place because nurses were not always present at a certain moment—even when a retrospective rationalisation of their actions took place in accordance with the rules (cf. Collins 1983, p. 186). The nurses’ adoption of this conservative behavioural position became apparent, as it was repeatedly reported to us, in the nurses’ tendency to fall back into their old behavioural patterns, even after the care strategies had apparently been clarified within the team and by the guidelines of the hospice management. This means that significant aspects of the daily routine of care work take place in an emergent context, despite the universally accepted rules. Should the emergent care procedure give rise to subjective maxims of behaviour, then the nurse will attempt to make them congruent with the rules, no doubt based on individual interpretations (cf. Collins 1975, p. 315).

### Conflicts from the structural perspective

In observing conflicts from the role perspective, it is possible to say that they result from varying interpretations of what might be considered as the correct form of care. If conflicts are examined from the structural perspective, however, a different explanatory model is necessary. Conflicts cannot be explained by examining individual acts of interpretation, but by transindividual structures. Accordingly, a different strategy must be used to manage conflicts when they become overly disruptive to the daily care routine. As a starting point, it is useful to recall Kurt Singer’s concept of conflict. He defines conflict as “a critical state of tension occasioned by the presence of mutually incompatible tendencies within an organismic whole, the functional continuity or structural integrity of which is thereby threatened” (Singer 1949a, p. 230). Whilst it would be too much to claim that the structural integrity of the hospice itself was under threat from a conflict situation, it is most certainly being challenged. We would therefore like to expand the concept of conflict, which has until now been defined as an oppositional, recurring positional difference,^10^ to include this aspect.

Singer suggests using the principle of integration as a possible method of handling conflicts. Integration means a structural change within which opposing positions are structurally mediated, in order to strengthen the institution as a whole. We were able to observe, during our study, that one desired outcome in the handling of a conflict was an increase in the degree of organisation by including interpersonal relationships (cf. Link 1979, p. 38). This was accomplished, for example, as mentioned by having palliative care doctors take over the essential medical care of the patients, to allow them to maintain a constant level of interaction with the care staff. Furthermore, the patients’ general practitioners were regularly encouraged to reconsider their attitudes as a result of their experiences in a hospice. The principle of integration can also be used in conflicts involving family members. Even when there is an attempt by a third party (the director of hospice, the director of patient care) to mediate in a conflict, the fundamental aim is always to attempt to involve the family members intensively in the terminal care their relatives are receiving. It is possible to speak of integration as a method of conflict regulation where care staff is regularly involved in the process of revising mission statements.

## Conclusion

One potential cause of conflict in the field of palliative care is an individual’s inability to “take the role of the other”. Here, the subjective interpretations of the rules governing the provision of care play are crucial, and they are constantly changing within the context of the social act. The effect of this is exacerbated due to differences in the areas of competence which are assigned, as well as by the generation of expertise which is distributed asymmetrically among the care staff (cf. Singer 1949b).^11^ Because the patients are individuals, the care they receive is in a constant state of renegotiation, so that it is only through this process that the nurses can actually identify the material content of the care and then try to match it to the rules and mission statements. A second possible cause of conflict was identifiable in situations which promoted conflict. If conflicts arise as a result of the implementation of rules, the hospice has two choices of how to proceed. It can tolerate the conflicts, as long as they are not disturbing the daily routine. This strategy tends to reinforce the conservative character of existing structures. A second, innovative, strategy is to increase the “degree of organisation” in the hospice in an attempt to better integrate the individuals concerned. Here, it was possible to observe how the nurses were involved in reformulating concepts of care and mission statements. In this instance, an attempt was being made to integrate divergent practical approaches to care, which the hospice saw as a “network of interpersonal influences” (Collins 1975, p. 298), by using a normative process.

We did not observe any actual conflict resolutions, where a conflict was resolved entirely. We only observed conflict regulation. On the one hand, allowances were made for conflicts to exist in the context of care staff adopting a role, and this situation was approved, or conflicts were regulated structurally by using integration processes. Further research needs to be undertaken to explore how a balance might be achieved between the two strategies for dealing with conflicts and to understand at which point the conservative strategy of tolerating conflicts gives way to the innovative strategy of structural change.

## Footnotes

1 According to the *Residential and Participation Law* [*Wohn*- *und Teilhabegesetz* (WTG)] in the German Land North Rhine Westphalia, any citizens residing in a hospice should be allowed, inter alia, “a life which can be conducted with the greatest possible degree of self-determination and independence”, “where their privacy and intimate space is protected”, “where they are accorded respect” and “where they can die in dignity” (§ 1 para. 2 WTG) These stipulations are based on the “Charter for People with Special Needs or Requiring Care” [“Charta der hilfe- und pflegebedürftiger Menschen”]. We are grateful to Mr Dirk Kassen at the Ministry of Health, Emancipation, Care and Age of North Rhine Westphalia for drawing our attention to the above.

2 In a previous study one of us examined the intrapersonal moral conflicts of hospice nurses and volunteers. Therefore our interest in this study is the interpersonal conflict. The basic question was: which conflict is relevant in practice. To be relevant a conflict consists if at least two parties have different, sometimes contradicting, exclusive positions concerning facts, an actual situation or circumstances. Such conflict has to be apparent otherwise it is not interpersonal.

3 Ethics Commission of the Medical Faculty of the Ruhr-University, Bochum, 21.10.2010, Registration-No.: 3850-10.

4 The interviews were recorded using a digital recording device after the interviewees had been informed and their written consent had been obtained. They were then transcribed verbatim. During this process the data were rendered anonymous. For the process of transcription, we used semi-interpretative working transcription [Halbinterpretative Arbeitstranskription] (HIAT) within which para-verbal and non-verbal expressions which are significant for the content are recorded; the score notation of transcription was implemented with the assistance of the EXMARaLDA computer programme. During the accompanying observations, handwritten notes were taken; after the meetings they were processed and rendered anonymous.

5 Therefore we have no category like “relationship between nurses and doctors/general practitioners” or “relationship among nurses” because the conflicts in these categories are always about the area of medication or care.

6 Tavor or Lorazepam is a benzodiazepine which is used for the treatment of anxiety and panic disorder. Properly used the sedation of the patient will be moderate. Lorazepam can lead to addiction.

7 There are also situations in which the patients know that they are going into a hospice but they do not know that they are destined to die there. They have an inaccurate understanding of the institution.

8 Ralf Dahrendorf writes: “Die Einstellung zu Konflikten, die im Gegensatz zur Unterdrückung und zur ‘Lösung’ Erfolg verspricht, weil sie den sozialen Realitäten angemessen ist, werde ich als *Regelung von Konflikten* bezeichnen. […] Konflikte verschwinden durch ihre Regelung nicht; sie werden nicht einmal notwendig weniger intensiv; in dem Maße aber, in dem es gelingt, sie zu regeln, werden sie kontrollierbar und wird ihre schöpferische Kraft in den Dienst einer allmählichen Entwicklung sozialer Strukturen gestellt.”

9 The same might be expected according to the theory of interaction ritual (cf. Erving Goffman 1971).

10 Using Singer’s work as a basis, Werner Link distinguished between conflicts on the one hand and positional differences, irreconcilable tendencies and competition on the other (cf. Link 1979, p. 43).

11 Singer reserves the use of the term “conflict” for cases involving differences “in which the collision threatens the stability and viability of the integrated unit” (Singer 1949b, p. 169).
